# The role of family and school-level factors in bullying and cyberbullying: a cross-sectional study

**DOI:** 10.1186/s12887-017-0907-8

**Published:** 2017-07-11

**Authors:** Leonardo Bevilacqua, Nichola Shackleton, Daniel Hale, Elizabeth Allen, Lyndal Bond, Deborah Christie, Diana Elbourne, Natasha Fitzgerald-Yau, Adam Fletcher, Rebecca Jones, Alec Miners, Stephen Scott, Meg Wiggins, Chris Bonell, Russell M. Viner

**Affiliations:** 1 UCL Institute of Child Health, Population, Policy and Practice Programme, 30 Guilford Street, WC1N 1EH, London, UK; 2 The University of Auckland, COMPASS, Auckland, New Zealand; 3 London School of Hygiene and Tropical Medicine, Clinical Trials Unit, Keppel Street, WC1E 7HT, London, UK; 4 University of Glasgow, MRC/CSO Social and Public Health Sciences Unit, Glasgow, UK; 5 UCL, Institute of Epidemiology & Health, 1-19 Torrington Place, London, WC1E 7HB -, London, UK; 6 Dpt. of Medical Statistics, London School of Hygiene and Tropical Medicine, Keppel Street, London WC1E 7HT, London, UK; 7 Cardiff University, School of Social Sciences, Cardiff, UK; 8 London School of Hygiene and Tropical Medicine, Faculty of Public Health and Policy, London, UK; 9 King’s College London, Institute of Psychiatry, Psychology and Neuroscience, London, UK; 10 UCL, Institute of Education, London, UK; 11 Department of Social and Environmental Health Research, Institute of Education, UCL, WC1H 9SH, London, UK; 12 UCL Institute of Child Health, Population, Policy and Practice Programme - General and Adolescent Paediatrics Unit, 30 Guilford Street (1st Floor), WC1N 1EH, London, UK

**Keywords:** Gatehouse bullying scale, Cyberbullying, Student-level variables, School-level variables, Multi-level models

## Abstract

**Background:**

Bullying and cyberbullying are common phenomena in schools. These negative behaviours can have a significant impact on the health and particularly mental health of those involved in such behaviours, both as victims and as bullies. This UK study aims to investigate student-level and school-level characteristics of those who become involved in bullying and cyberbullying behaviours as victims or perpetrators.

**Methods:**

We used data from 6667 Year 7 students from the baseline survey of a cluster randomized trial in 40 English schools to investigate the associations between individual-level and school-level variables with bullying victimization, cyberbullying perpetration, and cyberbullying victimization. We ran multilevel models to examine associations of bullying outcomes with individual-level variables and school-level variables.

**Results:**

In multilevel models, at the school level, school type and school quality measures were associated with bullying risk: students in voluntary-aided schools were less likely to report bullying victimization (0.6 (0.4, 0.9) *p* = 0.008), and those in community (3.9 (1.5, 10.5) *p* = 0.007) and foundation (4.0 (1.6, 9.9) *p* = 0.003) schools were more likely to report being perpetrators of cyberbullying than students in mainstream academies. A school quality rating of “Good” was associated with greater reported bullying victimization (1.3 (1.02, 1.5) *p* = 0.03) compared to ratings of “Outstanding.”

**Conclusions:**

Bullying victimization and cyberbullying prevalence vary across school type and school quality, supporting the hypothesis that organisational/management factors within the school may have an impact on students’ behaviour. These findings will inform future longitudinal research investigating which school factors and processes promote or prevent bullying and cyberbullying behaviours.

**Trial registration:**

Trial ID: ISRCTN10751359 Registered: 11/03/2014 (retrospectively registered).

## Background

Bullying is defined as repeated and harmful behaviour, characterised by a strong imbalance of power between the bully and the victim [1]. Involvement in bullying behaviours is a widespread phenomenon in childhood and adolescence that can have a negative impact on health such as later anxiety problems [2], depression and self-harm [3, 4], antisocial behaviour [5], and suicide or attempted suicide [6, 7], as well as substance misuse and poor educational outcomes [8].

Researchers have investigated the characteristics of adolescents who are involved in bullying behaviours. In a meta-analysis of 28 studies, Tippett and Wolke [9] found a significant but weak association between low socio-economic status (SES) and being a victim of bullying. Bullying behaviours differ across sex and ethnicities. For example, males have been found to engage in bullying behaviours that are more physical in nature (such as hitting or kicking other classmates) than females, who seem to exhibit more relational bullying behaviours (such as excluding classmates or spreading rumours about them) [10]. A study conducted in the UK [11] highlighted differences in bullying subtypes across ethnicities, with Pakistani and Caribbean girls more often being perpetrators of bullying than girls in other ethnic groups.

There is evidence that characteristics of the school can also influence bullying. For example, the size of the school [12] and the neighbourhood in which the school is located [13, 14] have been associated with bullying behaviours, with schools with a large number of students showing higher proportions of bullying behaviours, and low school/neighbourhood SES being associated with higher rates of bullying behaviours. However, we do not know the relationship between other school-level factors and bullying behaviours.

Recently, a new form of bullying has emerged, labelled “cyberbullying,” which is defined as an aggressive act carried out by a single individual or a group of individuals using electronic forms of contact [15]. Research on cyberbullying is at an early stage but we know that the experience of being cyberbullied is very distressing [16].

The family and socio-demographic characteristics of those who engage in cyberbullying have been little studied and most data currently available come mainly from the USA [17].

The aim of this exploratory study is to fill the gaps highlighted above by investigating a range of school characteristics that may be associated with 1) bullying victimization and 2) cyberbullying (victimization and perpetration). Understanding whether and what school-level characteristics have an impact on students’ behaviour is particularly important to guide and implement intervention programs that target schools.

## Methods

We used data from the baseline survey of the INCLUSIVE study, a cluster randomized controlled trial of an intervention aimed at reducing bullying and aggressive behaviours in 11 to 16 year old students in secondary schools. Baseline data were collected before randomization in May–June 2014 from all Year 7 classes (age 11–12 years) in 40 participating secondary schools within the state education system across south-east England. Full details of the sampling methodology are available in the study protocol [18]. Schools exclusively for those with learning disabilities, behaviour problems (e.g. student referral units) and very poorly performing schools with an Ofsted rating of “Inadequate” were not included in the sample [18]. Data were collected through questionnaires completed in school in confidential sessions supervised by the research team. A total of 6667 students provided baseline data. Students provided demographic details by self-report. Other student-level outcome measures were also assessed by self-report as follows.

### Bullying measures

Bullying victimization was assessed using the Gatehouse Bullying Scale (GBS), a 12-item short and reliable instrument previously used in school-based surveys and shown to be related to other measures of social attachments, school engagement, and anxiety and depressive symptoms [19]. The GBS enquires about four categories of bullying, i.e. being the subject of recent name calling, rumours, being left out of things, and physical threats or actual violence from other students in the last three months. In each of these, questions ask about the recent experience of that type of bullying (yes or no), how often it occurred (most days, about once a week, less than once a week), and how upset the student was by each type of bullying (from “I was quite upset,” “a bit upset” to “not at all”). We combined frequency and distress responses to calculate GBS scores as follows: 0 = Not bullied; 1 = Bullied, but not frequently, and not distressed by it; 2 = Bullied, either frequently or distressed, but not both; and 3 = Bullied frequently and distressed. We used these to define two categories of bullying: bullying victimization (GBS score of 2 or 3 collapsed together indicating either frequent or distressing bullying or both) or severe bullying (GBS score of 3 indicating frequent distressing bullying).

The GBS does not specifically include or exclude bullying through social media or other online activities. Cyberbullying was specifically assessed using two items adapted from Smith and colleagues’ DAPHNE II questionnaire [15] asking whether the participant was bullied (victim) and/or bullied someone else (perpetrator) through mobile phone use or the internet over the past three months. Responses were on a five-point Likert scale for each question, from 1 = No, I have not; 2 = Yes, once or twice; 3 = Yes, two or three times a month; 4 = Yes, about once a week; to 5 = Yes, several times a week or more. We dichotomised responses for these analyses into “not/rarely bullied” and “bullied/frequently bullied” for victims and “not/rarely bullied others” and “bullied/frequently bullied others” for perpetration (by collapsing responses 1 and 2 together and 3, 4 and 5 together to obtained these two categories for both cyberbullying victimization and perpetration).

### Other student-level characteristics

Young people provided data on SES and family composition. Socioeconomic status was assessed using the Family Affluence Scale (FAS), developed specifically for reporting of SES by young adolescents [20]. Four questions assess car ownership, children having their own bedroom, the number of computers at home, and the number of holidays taken in the past 12 months. A composite FAS score is calculated for each student based on his or her responses to these four items. For our analyses, scores were collapsed to give FAS tertiles of low, medium, and high family affluence, where FAS low (score = 0, 1 and 2) indicates low affluence, FAS medium (score = 3, 4 and 5) indicates middle affluence, and FAS high (score = 6, 7, 8 and 9) indicates high affluence.

Family composition was assessed based on student reports of who lived in their house with them. To create a dichotomous variable (two parents vs lone parent), students were classified as having two parents if they reported living with any two of the following: mother, father, step-mother, step-father, foster mother, and foster father. Students were classified as having a lone parent if they reported living with only one of these parents. In our sample, 73.91% of students reported living with two parents.

### School characteristics

Data were available on the following school-level characteristics:

1. School-level deprivation:

- Proportions of students eligible for free school meal (FSM): this is a widely used proxy measure for economic deprivation in the UK [21, 22]. In England and Wales, local education authority-maintained schools must provide a free midday meal to students if they or their parents receive specific benefits. We used the percentage of students eligible for FSMs at any time during the past six years, obtained from publicly accessible data from Department of Education school performance Tables [23]. The proportion of students eligible for FSM in our sample schools ranged from 3.0% to 79.2% (mean = 36.4%, SD = 19.6).
- The Income Deprivation Affecting Children Index (IDACI) score of the schools’ postal address: the IDACI scores deprivation that measures the proportion of children in a small area under the age of 16 who live in low income households. It is supplementary to the Indices of Multiple Deprivation and is used for calculation of the educational contextual value added score, measuring children’s educational progress [24].

2. School type: Our sample includes of five different types of schools: community (*n* = 5), where premises and funding are provided by local authorities; foundation (*n* = 6), where the school owns its own premises but funding comes from the local authority; voluntary-aided (*n* = 4), where the premises are owned by a charity but funding is at least partly from the local authority; sponsor-led academy (*n* = 6) which are usually created from an underperforming school which obtained an independent business or charitable sponsor and where funding comes directly from central government; and converter academy mainstream (*n* = 18), which are successful schools which have opted to gain more autonomy and have funding directly from central government [25]. Voluntary-aided, community and foundation schools follow the National Curriculum and are supervised by the local authority. In our sample, all voluntary-aided schools were faith schools. Academies do not have to follow the National Curriculum except for core subjects. In addition, they have more freedom in setting their own term times and changing the length of school days.
3. School size: the total number of students in the school [26].
4. Sex composition: mixed sex or single sex [26].
5. School quality:

- Most recent overall Ofsted rating: in England, schools are inspected by a statutory body, the Office for Standards in Education, Children’s Services and Skills [26]. Ofsted inspections are carried out every 2–5 years, depending on inspection outcomes [27] and all schools had data from 2011 to 14. Schools were classified as 1 = “Outstanding”, 2 = “Good”, 3 = “Requires improvement” or 4 = “Inadequate” based on the quality of teaching, leadership and management, achievement of students, and behaviour and safety of students at the school. Our sample included no schools with a rating of “Inadequate.”
- Value added (VA) score: a second school quality rating was the VA score, an official measure of the progress students make between different stages of education. To calculate this, a median line approach is used whereby the VA score for each student is the difference (positive or negative) between their own output point score (end of Key Stage 4) and the median output point score achieved by others with the same or similar starting point (Key Stage 2 or 3), or input point score [23]. Scores for VA were given, with schools that neither added nor subtracted value being given a score of 1000.

### Statistical analysis

We first described the frequency of bullying (collapsing GBS scores in two different ways to obtain both “significant” and “severe bullying” with only significant bullying being used for subsequent analyses), and cyberbullying perpetration and victimization by sex and ethnicity among students and the distribution of school-level factors across schools.

Previous research has shown that individual-level factors considered here (gender, ethnicity, SES and family composition) have been consistently associated with mental health and bullying outcomes. Therefore, all the models in our study included these factors as covariates.

In step one we examined the association of each school-level factor with bullying and cyberbullying outcomes in separate models, adjusted for all individual-level factors and took account of clustering at the school level. In step two we used multilevel mixed effects logistic regression to examine the associations between bullying and cyberbullying outcomes and individual- and school-level factors, with a random effect for school. This final model included all school-level variables that were found significant at the *p* < 0.1 in step two, together with all individual-level factors.

Interactions were tested between all individual- and school-level factors that were significant at the 10% level in the final multivariable model. Data were analysed using STATA 13.0 (College Station, TX).

## Results

Data were available for 6667 (3103 males, 47%) Year 7 students (mean age = 11.8, SD = 0.4) in 40 schools in south-east England. Of these, 39.4% were White British, 25.0% were Asian or Asian British, 14.0% were Black or Black British, 8.5% were White (other), 7.0% reported having mixed ethnicity, 1.0% were Chinese or Chinese British, and 5.1% were from other ethnic groups.

The distribution of bullying victimization (either frequent or distressing) and severe bullying categories and cyberbullying (victimization and perpetration) by sex and ethnicity are shown in Table 1.

**Table 1:** Prevalence of bullying victimization and cyberbullying by sex and ethnicity

**GBS measures of bullying victimization**														
Bullying victimization either frequent or distressing	Whole sample		White British		White Other		Asian/Asian British		Black/Black British		Mixed Ethnicity		Other Ethnic group	
	*N*	%	*n*	%	*n*	%	*n*	%	*n*	%	*n*	%	*n*	%
Male	948	32.1	350	30.33%	89	33.71%	261	32.42%	122	31.44%	72	35.82%	54	38.57
Female	1262	38.16	528	38.68%	108	39.42%	270	35.67%	191	41.70%	96	41.20%	69	31.36
Severe bullying victimization														
	Whole sample		White British		White Other		Asian/Asian British		Black/Black British		Mixed Ethnicity		Other Ethnic group	
	*N*	%	*n*	%	*n*	%	*n*	%	*n*	%	*n*	%	*n*	%
Male	295	9.99%	98	8.49%	31	11.74%	93	11.55%	28	7.22%	23	11.44%	22	15.71%
Female	450	13.61%	168	12.31%	45	16.42%	95	12.55%	80	17.47%	33	14.16%	29	13.18%
**Cyberbullying measures**														
Cyberbullying perpetration	Whole sample		White British		White Other		Asian/Asian British		Black/Black British		Mixed Ethnicity		Other Ethnic group	
	*N*	%	*n*	%	*n*	%	*n*	%	*n*	%	*n*	%	*n*	%
Male	34	1.13	8	0.68%	6	2.23%	5	0.61%	6	1.53%	9	4.50%	0	0
Female	15	0.45	6	0.44%	1	0.35%	2	0.26%	4	0.84%	0	0.0%	2	0.89%
Cyberbullying victimization														
	Whole sample		White British		White Other		Asian/Asian British		Black/Black British		Mixed Ethnicity		Other Ethnic group	
	*N*	%	*n*	%	*n*	%	*n*	%	*n*	%	*n*	%	*n*	%
Male	58	1.94	15	1.28%	9	3.38%	17	2.10%	11	2.78%	4	1.96%	2	1.44%
Female	150	4.48	71	5.18%	15	5.38%	23	3.00%	23	4.88%	7	2.95%	11	4.95%

The table shows number and percentage of students who reported bullying victimization (frequent or distressing) and severe bullying (frequently and distressing, cyberbullying of others (cyberbullying perpetration) and those who report having frequently been victims of cyberbullying (Cyberbullying victimization)

### Student-level variables

The associations of individual-level variables with bullying outcomes are shown in the final multivariable model. Sex was strongly associated with all bullying outcomes: girls were more likely to be significantly bullied and cyberbullied but less likely to be cyberbullies. Bullying victimization or cyberbullying victimization did not vary across ethnic groups when adjusted for all other factors. However, students of mixed ethnicity were more likely to be cyberbullying perpetrators than white British students. Individual-level deprivation (low compared to medium family affluence) was associated with greater risk of being a cyberbullying victim or perpetrator. Independently of deprivation, young people from a single parent household were more likely to be bullied and cyberbullied compared to those coming from a two-parent household.

### School-level variables

Characteristics of schools are shown in Table 2 with further detail on student characteristics by type of school shown in Table 5 (in the Appendix). Associations between each school-level factor and bullying and cyberbullying outcomes, adjusted for all individual-level factors, were tested in separate models and results are shown in Table 3. All those school-level factors that were found to be significant at the 10% level were included in our final model, where all individual-level factors were again included. Results of this model are shown in Table 4.

**Table 2:** Characteristics of 40 schools and their students

Free School Meal	Mean	SD	Min	Max		
FSM value	34.9	20.3	3	79.2		
Sex	Students N	Students %			School N	School %
Mixed sex	5055	75.8			30	75
Single sex F	1175	17.6			7	17.5
Single sex M	437	6.5			3	7.5
School size	Mean	SD	Min	Max		
Total number of students	1157.7	301.8	504	1841		
Type of school	Students N	Students %			School N	School %
Converter - Academy Mainstream	3320	49.8			19	47.5
Voluntary aided	452	6.8			4	10
Community schools	883	13.2			5	12.5
Academy - sponsor led	824	12.4			6	15
Foundation school	1188	17.4			6	15
IDACI	Mean	SD	Min	Max		
IDACI score	24.2	20.0	0	69.8		
Ofsted	Students N	Students %			School N	School %
Outstanding	2116	32.1			11	27.5
Good	3907	59.4			25	62.5
Requires Improvement	559	8.4			4	10
Value Added Score	Mean	SD	Min	Max		
VA Value	1005.0	20.4	921.3	1040.5		

NB. Data are shown for all 40 schools except where noted

**Table 3:** Partially adjusted associations of school-level factors with bullying outcomes

	Significant victimization			Cyberbullying perpetration			Cyberbullying victimization		
	adjusted OR	95% CI	*p* value	adjusted OR	95% CI	*p* value	adjusted OR	95% CI	*p* value
Free School Meal									
	1.00	.9955028 1.00949	0.5	1.02	1.001606 1.045892	*0.03*	1	.9961553 1.017999	0.2
School sex									
Mixed sex	1			1			1		
Single sex F	.73	.5643272 .9515022	*0.02*	.72	.1746976 2.94945	0.6	.9	.5423573 1.501808	0.7
Single sex M	1.21	.8328359 1.765787	0.3	.43	.071117 2.576839	0.3	.84	.308904 2.275078	0.7
School size									
Total number of students	1	.9943321 1.000767	0.1	.99	.9812291 1.00784	0.4	1	.9945173 1.00771	0.7
Type of school									
Converter - Academy Mainstream	1				1			1	
Voluntary aided	.69	.4772175 .9991611	*0.05*	.49	.0571277 4.354682	0.5	.72	.3356624 1.567076	0.4
Community schools	1.04	.7711656 1.409772	0.8	4.25	1.543457 11.71325	*0.005*	1.63	.9985184 2.66383	0.05
Academy - sponsor led	.86	.6425759 1.165844	0.3	2.10	.687105 6.43157	0.2	.55	.279567 1.076904	0.08
Foundation school	.88	.6629961 1.162673	0.4	4.73	1.827836 12.25967	*0.001*	.98	.5895541 1.61874	0.9
IDACI									
IDACI score	1	.9960809 1.006945	0.6	1.01	.9956466 1.038245	0.1	1.43	1.030389 1.977603	0.2
Ofsted									
Outstanding	1			1			1		
Good	1.28	1.024871 1.596348	*0.03*	1.94	.7071846 5.335933	0.2	1.03	.6593583 1.609838	0.9
Requires Improvement	1.3	.9037564 1.872556	0.1	4.01	1.054254 15.24303	*0.04*	.93	.4169812 2.062901	0.8
Value Added Score									
VA Value	1	.9918419 1.001285	0.1	.99	.9743484 1.01292	0.5	1	.994339 1.01547	0.4

**Table 4:** Multilevel models of associations of bullying victimization, cyberbullying perpetration, and cyberbullying victimization with individual- and school-level factors

	Significant bullying			Cyberbullying perpetration			Cyberbullying victimization		
	adjusted OR	95% CI	*p* value	adjusted OR	95% CI	*p* value	adjusted OR	95% CI	*p* value
Sex									
Male	1			1			1		
Female	1.43	1.26206 1.619914	*<0.000*	0.38	.1972747 0.7490761	*0.004*	2.31	1.655916 3.226372	*<0.000*
Ethnicity									
White British	1			1			1		
White other	1.16	.940071 1.425895	0.2	2.08	.766005 5.651757	0.1	1.38	.8434737 2.254222	0.2
Asian/Asian British	0.99	.8358366 1.164221	0.9	0.77	.2594833 2.313221	0.6	0.81	.5255289 1.254694	0.3
Black/Black British	1.17	.965876 1.407702	0.1	1.99	.7520412 5.260238	0.2	1.16	.7219747 1.852542	0.5
Mixed ethnicity	1.25	.9936084 1.565785	0.06	3.06	1.142718 8.189537	*0.03*	0.64	.3147527 1.314576	0.2
Other	1.02	.7956576 1.323007	0.8	0.44	.0543722 3.594754	0.4	1.11	.5857842 2.113013	0.7
Family structure									
Two parents	1			1			1		
Single parent	1.19	1.044057 1.35345	*0.009*	1.01	.5154379 1.982387	0.1	1.44	1.042272 2.002339	*0.03*
Socio-economic status									
FAS medium	1			1			1		
FAS low	1.34	.9822942 1.834434	0.06	3.85	1.182666 12.54253	*0.02*	2.31	1.197309 4.476614	*0.01*
FAS high	1.09	.9617164 1.229097	0.2	1.82	.8781477 3.764663	0.1	1.37	.9698386 1.935498	0.07
Free School Meal									
	Significant victimization			Cyberbullying perpetration			Cyberbullying victimization		
School sex	adjusted OR	95% CI	*p* value	adjusted OR	95% CI	*p* value	adjusted OR	95% CI	*p* value
Mixed sex	1								
Single sex F	0.79	.6177481 1.005942	0.056						
Single sex M	1.38	.9574116 1.987917	0.08						
School size									
Total number of students									
Type of school									
Converter - Academy Mainstream	1			1			1		
Voluntary aided	0.63	.4443628 .884386	*0.008*	0.50	.0595084 4.179261	0.5	0.72	.3356624 1.567076	0.4
Community schools	0.97	.7399493 1.278299	0.8	3.89	1.447156 10.45903	*0.007*	1.63	.9985184 2.66383	*0.05*
Academy - sponsor led	0.78	.5902065 1.044216	0.1	2.49	.8481433 7.335429	0.1	0.55	.279567 1.076904	0.08
Foundation school	0.91	.7016586 1.172529	0.4	3.95	1.571424 9.932699	*0.003*	0.98	.5895541 1.61874	0.9
IDACI									
IDACI score									
Ofsted									
Outstanding	1			1					
Good	1.26	1.021447 1.546847	*0.03*	1.61	.6172557 4.190908	0.3			
Requires Improvement	1.12	.8000499 1.58324	0.5	2.79	.8696668 8.973181	0.08			
Value Added Score									
VA Value									

The table shows the final model with intra-class correlation coefficients, student-level factors, and school-level factors associated to bullying victimization and cyberbullying outcomes

Adjusted school-level intra-class correlation coefficients ranged from 0.19 to 0.25 for each of our outcome measures.

## Discussion

This is the first study to examine both student- and school-level factors associated with bullying victimization and cyberbullying in a large sample of English young people. We found that 32% of boys and 38% of girls report bullying victimization (either frequent or distressing, or both) with 10% of boys and 14% of girls reporting severe bullying (frequent and distressing) over the same period. Bullying through online methods (cyberbullying victimization) was reported by 2% of boys and 4.5% of girls, with 1% of boys and 0.5% of girls reporting being cyberbullies. Bullying of all types varied significantly by school, although the school-level explained only 1.9% to 2.5% of the variance of bullying across the sample.

Bullying victimization and cyberbullying victimization were reported more often by girls. A potential explanation for this result is that girls are more likely to be exposed to bullying from both other girls and boys as well (i.e. with the latter engaging in more gender-based violence such as sexual harassment or sex-based jokes), as opposed to boys, who might be bullied primarily by other boys and less likely to be bullied by girls. Therefore, this may reflect an actual difference in terms of frequency and emotional distress associated with victimization across gender in our sample. Another potential explanation is that girls are more likely to report actual frequency and levels of distress associated with victimization compared to males, perhaps as a consequence of less shame associated with reporting being bullied compared to boys, but this remains highly speculative. We found minimal association between bullying and cyberbullying and ethnicity, aside from a low significance association between mixed ethnicity and cyberbullying perpetration. This result adds to a scenario of mixed findings, where the relationship between ethnicity and peer aggression is still unclear. Those from more deprived families were more likely to be victims of cyberbullying but also cyberbullying perpetrators. Students with a lone parent were found to be more likely to be bullied and cyberbullied. The school-level factors independently associated with bullying risk were school quality rating and school type. An Ofsted rating of “Good” was associated with higher risk of significant bullying compared with schools rated “Outstanding”. These findings were independent of other school-level factors including school type.

This finding suggests that school organisations that perform well in terms of leadership and management engender school climates that are protective against bullying. We recognise that Ofsted ratings take into account a number of aspects of a school aside from leadership and management, including school ethos, awareness of bullying behaviours and how to prevent and manage them. We speculate that these latter aspects of Ofsted are particularly important in explaining why students in “Outstanding” schools report lower bullying victimization scores compared to schools with an Ofsted rating of “Good”. We recognise that school level factors other than those measured in the present study are likely to influence bullying behaviours (i.e. staff health and well-being). Note that the lowest Ofsted rating (“Requires improvement”) was not associated with bullying risk, possibly due to the small number of schools in this category.

Students in voluntary-aided schools (faith schools in our sample) were less likely to be bullied compared to those in the largest group of schools, i.e. mainstream state schools that had recently converted to academy status. This supports the notion that elements of school ethos and culture are protective against bullying. Alternative hypotheses include that faith schools might attract students from families in whom bullying is less common or where children are more resilient to being bullied. In contrast, cyberbullying perpetration was more common amongst community and foundation schools compared to converter academy mainstream schools. The reasons for this are unclear and need to be investigated further in longitudinal samples. We did not find that the sex intake of a school was strongly associated with bullying, although an interaction between family affluence and school sex suggested that the association of high SES with lower risk of bullying is stronger in boys-only schools than in girls-only or mixed schools.

### Strengths and limitations

We used baseline data from a very large school-based survey with a sample purposively recruited to be representative of the range of state schools in England, with the exception of schools rated “Inadequate.” Response rates were high. We used validated outcomes for bullying and cyberbullying and accepted measures of school type and quality. Analyses accounted for clustering of data at the school-level.

Our data are subject to a number of limitations. Data are cross-sectional and causality cannot therefore be inferred. We had limited power to examine smaller ethnic groups and less common school types. This study is limited by the data collected in the Inclusive trial, which did not include a separate measure of non-cyberbullying perpetration.

The extent to which more traditional measures of bullying such as the GBS also pick up elements of cyberbullying is unclear.

Finally, we accept that the study was not powered for interaction tests and furthermore we have carried out a large number of tests leading to an increased risk of type I error.

## Conclusions

We investigated whether school-level factors influence bullying and cyberbullying in a large sample of secondary school students. School type and school quality measures were associated with bullying risk. These preliminary findings pave the way for future research investigating which school factors and processes promote or prevent bullying and inform development of interventions to prevent bullying and cyberbullying in schools.

## Appendix

**Table 5:** Student characteristics by school type

	Gender		Ethnicity						Lone		FAS		
School Type	M	F	WB	WO	A/AB	B/BB	ME	OE	YES	NO	LOW	MED	HIGH
	%	%	%	%	%	%	%	%	%	%	%	%	%
Voluntary aided	57	43	30	14	7	29	12	7	34	66	3	30	67
Community Schools	40	60	35	11	22	17	9	6	33	67	4	43	53
Converter Academy–mainstream	47	53	51	7	26	7	4	4	21	79	2	28	70
Academy Sponsor-led	56	44	29	9	11	30	11	9	36	64	5	40	56
Foundation schools	43	57	20	9	41	13	7	9	25	75	5	40	54

Ethnicity groups: *WB* White British, *WO* White Other, *A/AB* Asian/Asian British, *B/BB* Black, black British, *ME* Mixed Ethnicity, *OR* Other ethnic group

## Abbreviations

FAS: Family Affluence Scale
FSM: Free School Meal
GBS: Gatehouse Bullying Scale
IDACI: Index Deprivation Affecting Children Index
OFSTED: Office for Standards in Education, Children’s Services and Skills
VA: Value Added Score
